# Genome-Wide Identification of R2R3-MYB Transcription Factor and Expression Analysis under Abiotic Stress in Rice

**DOI:** 10.3390/plants11151928

**Published:** 2022-07-25

**Authors:** Lihua Kang, Yangyang Teng, Qiwen Cen, Yunxia Fang, Quanxiang Tian, Xiaoqin Zhang, Hua Wang, Xian Zhang, Dawei Xue

**Affiliations:** 1 College of Life and Environmental Sciences, Hangzhou Normal University, Hangzhou 311121, China; kanglihua_hznu@163.com (L.K.); tyyhznu@163.com (Y.T.); cenqiwen@stu.hznu.edu.cn (Q.C.); yxfang12@163.com (Y.F.); quanxiang@hznu.edu.cn (Q.T.); zxq@hznu.edu.cn (X.Z.); 2 State Key Laboratory for Managing Biotic and Chemical Threats to the Quality and Safety of Agro-Products, Institute of Virology and Biotechnology, Zhejiang Academy of Agricultural Sciences, Hangzhou 310021, China; wanghua3@hotmail.com

**Keywords:** R2R3-MYB, genome-wide identification, abiotic stress, rice

## Abstract

The myeloblastosis (MYB) family comprises a large group of transcription factors (TFs) that has a variety of functions. Among them, the R2R3-MYB type of proteins are the largest group in plants, which are involved in controlling various biological processes such as plant growth and development, physiological metabolism, defense, and responses to abiotic and biotic stresses. In this study, bioinformatics was adopted to conduct genome-wide identification of the R2R3-MYB TFs in rice. We identified 190 MYB TFs (99 R2R3-MYBs), which are unevenly distributed on the 12 chromosomes of rice. Based on the phylogenetic clustering and protein sequence characteristics, OsMYBs were classified into five subgroups, and 59.6% of the *Os2R_MYB* genes contained two introns. Analysis of cis-acting elements in the 2000 bp upstream region of *Os2R_MYB* genes showed that all *Os2R_MYB* genes contained plant hormones-related or stress-responsive elements since 91.9%, 79.8%, 79.8%, and 58.6% of *Os2R_MYB* genes contain ABRE, TGACG, CGTCA, and MBS motifs, respectively. Protein–protein network analysis showed that the *Os2R_MYBs* were involved in metabolic process, biosynthetic process, and tissue development. In addition, some genes showed a tissue-specific or developmental-stage-specific expression pattern. Moreover, the transcription levels of 20 *Os2R_MYB* genes under polyethylene glycol (PEG) and cadmium chloride (CdCl_2_) stress inducers were dissected by qRT-PCR. The results indicated genes with an altered expression upon PEG or CdCl_2_ stress induction. These results potentially supply a basis for further research on the role that *Os2R_MYB* genes play in plant development and stress responses.

## 1. Introduction

MYB transcription factor families are abundant in plants, accounting for about 9% of total transcription factors in *Arabidopsis thaliana* [1]. Proteins differ on the number of basic MYB domain repeats, conferring the DNA-binding capacity. A MYB DNA-binding domain is about 52 amino acid residues (R) long. Approximately 18–19 amino acid residues regularly repeat in the tryptophan residue, forming a helix-turn-helix (HTH) fold [2,3]. On the basis of the number of adjacent repeats, MYB can be classified into four categories: R1/R2, R3-MYB (1R-MYB), R2R3-MYB (2R-MYB), R1R2R3-MYB (3R-MYB), and 4R-MYB, which contain one, two, three, and four MYB repeats, respectively. In plants, most MYB transcription factors belong to the R2R3 type [4,5].

MYB proteins function in a variety of biological processes, including plant developmental regulation, plant hormone responses, cell differentiation, and adaption to biotic and abiotic stresses. The *MYB* gene regulating anthocyanin biosynthesis was firstly identified in *Zea mays* (*C1*) [6]. Since then, numerous *MYB* genes have been isolated and identified in plants. For example, expression of the snapdragon (*Antirrhinum majus*) R2R3-MYB proteins AmMYB308/330 downregulated the expression level of the genes related to lignin synthesis in tobacco, resulting in lignin reduction [7]. Co-expression of soybean *GmIFS1* (encoding isoflavone synthase 1) and Arabidopsis *AtMYB12* in tobacco can effectively promote isoflavone synthesis [8]. In strawberry, the natural variation of *MYB10* caused the change in color of skin and flesh [9]. The R2R3-MYB TF MYB6 regulates different branches of the phenylpropanoid biosynthesis pathway and promotes anthocyanin biosynthesis in *Populus tomentosa* [10]. Some MYB TFs have also been comprehensively studied in abiotic stress. Transgenic Arabidopsis plants over-expressing the *OsMYB3R-2* showed enhanced resistance to drought, salt, and cold stress [11]. Similarly, enhanced cold-stress tolerance was observed in tobacco overexpressing the *PbrMYB5* [12], and transgenic Arabidopsis plants overexpressing the *MYB4* exhibited higher cadmium (Cd) tolerance [13]. The R2R3-MYB TFs in rice exerted an important effect in plant development and abiotic stress response. In development, some MYB TFs have been reported to affect male fertility [14,15], hull and grain development [16], and flower and spikelet development [17] of rice. In addition, it also showed a significant effect on salt [18], drought [19], and cold [20] resistance and heavy metal [21] tolerance in rice. Studies have shown that MYB-binding region enrichment was related to drought induction, suggesting its regulatory role in rice drought response [22].

Rice (*Oryza sativa* L.), as the most important food crop in China, provides the staple food for about 50% of the world’s population. It ranks first in China’s food production chain, but the yield is easily affected by the availability of water, temperature, diseases, and pests during its development. With the development of modern production, soil cadmium pollution is emerging as a more serious problem. Since rice contains transporters uptaking cadmium, a serious threat has been raised up to food safety and human health [23]. However, the studies about the MYB transcription factor involved in heavy metal stress are rarely reported. Therefore, it is very important to study the mechanism underlying the stress responses of rice varieties. In this study, the polyethylene glycol 6000 (PEG) and cadmium chloride (CdCl_2_) treatments were applied to simulate drought and heavy metal stress for exploring its related response pathways. The availability of rice genome sequencing has facilitated widespread biological research [24,25]. In this study, all rice MYB proteins in the genome database were identified using bioinformatics methods, including chromosome localization, phylogenetic analysis, gene structure analysis, and promoter cis-element analysis of *Os2R_MYBs*. In addition, based on genetic relevance, predicted gene expression levels, and physicochemical properties of proteins, twenty 2R- (R2R3-) *OsMYB* genes were selected, and their expression was analyzed after PEG and CdCl_2_ treatments on Nipponbare. Quantitative real-time (qRT)-PCR was used to determine gene transcription levels and the expression pattern in response to abiotic stress. This study can be regarded as a basis for functional research on the MYB TF family in rice, which will be beneficial to improving the quality and yield of rice and breeding new varieties.

## 2. Results

### 2.1. Genome-Wide Identification and Chromosomal Locations of OsMYBs

**Figure 1:**
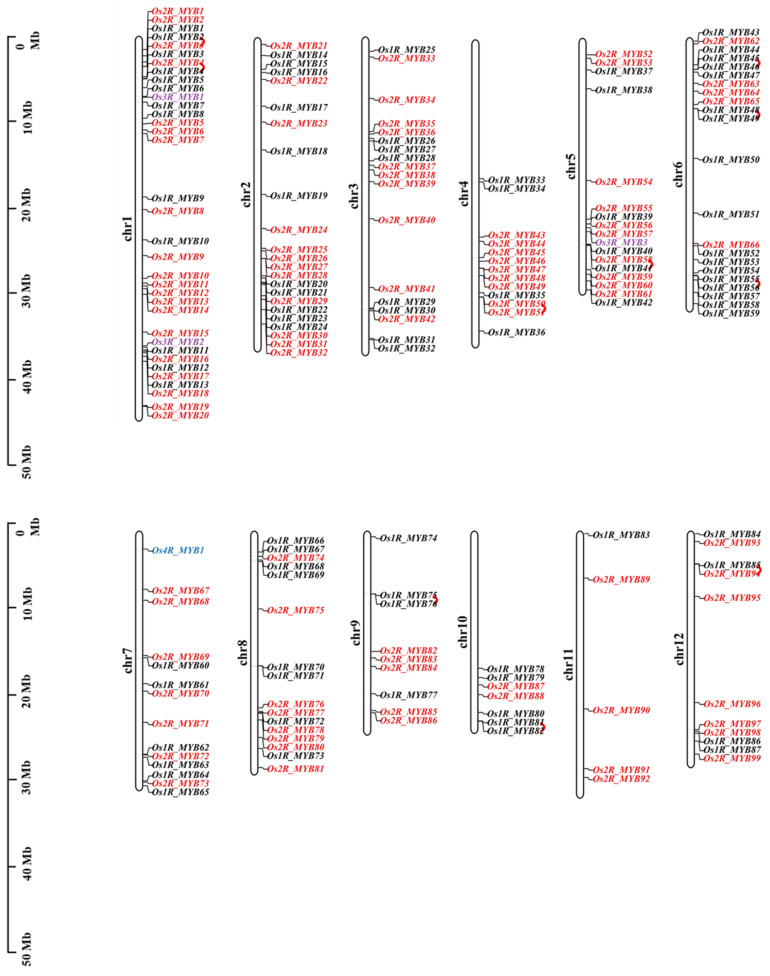
Distribution of *OsMYBs* on rice chromosomes. The rod represents the 12 chromosomes of rice, and the scale on the left indicates its length. Different colors represent the different MYB type: 1R_MYB (Black); 2R_MYB (Red); 3R_MYB (purple); 4R_MYB (blue). The repeat gene pairs were linked using red curves.

In this study, 190 *OsMYB* genes were identified by genome-wide analysis and classified into four subfamilies according to the number of MYB DNA-binding domains: 1R-MYB (R1/R2, R3-MYB), 2R-MYB (R2R3-MYB), 3R-MYB (R1R2R3-MYB), and 4R-MYB. We found that R2R3-MYB genes accounts for 52.1% of the total MYB genes in rice. Studies have revealed that most of the MYB TFs involved in resistance to adversity are the R2R3-MYB type [26]. Based on the number of adjacent repeats, they were named as Os1R_MYB1~Os1R_MYB87, Os2R_MYB1~Os2R_MYB99, Os3R_MYB1~Os3R_MYB3, and Os4R_MYB1. The results of chromosome visualization (Figure 1) showed that 190 *OsMYB* genes are located on 12 chromosomes. There were more *OsMYB* genes on chromosomes 1, 2, and 6 (35, 23, and 22, respectively) and fewer *OsMYB* genes on chromosomes 9, 10, and 11 (9, 7, and 5, respectively). There were 20 *Os2R_MYB* genes accounting for 20.2% of the total 2R-MYB-type genes located on chromosome 1. In addition, there were 10 repeat gene pairs. We also analyzed the physicochemical properties of the family member 2R-MYB proteins. The results (Table S1) show that the GRAVY of 99 proteins was negative, and average instability index was 57.15, indicating that *Os2R_MYB* proteins were almost all unstable hydrophilic proteins (GRAVY < 0, instability index > 40).

### 2.2. Phylogenetic Analysis of OsMYBs

**Figure 2:**
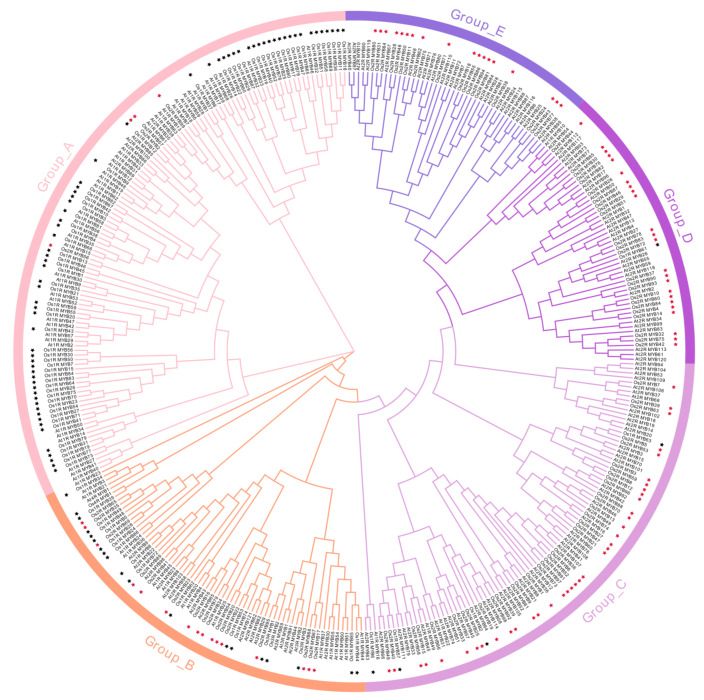
Phylogenetic relationship of *Oryza sativa* L. and *Arabidopsis* MYB proteins. The phylogenetic tree of MYB proteins was constructed by the neighbor-joining method of Mega-X with a bootstrap value set to 1000. It was classified into five groups (Group_A/B/C/D/E) according to the sequence characteristic of proteins. The asterisk indicates a protein origin from rice, and the crimson asterisk represents R2R3_MYB proteins.

In plants, the R2R3-MYB type of proteins play an important role in the developmental stages and under the stress responses. Therefore, phylogenetic trees of MYB proteins in *Oryza sativa* L. and *Arabidopsis thaliana* were constructed (Figure 2) to further study the evolutionary relationships between 2R-MYB proteins. Multiple sequence alignment of rice 2R-MYB proteins (Supplementary Figure S1) showed the “-W-(X18/X19)-W-(X18/X19)-W-…-W/F/I/L-W(X18/X19)-W-” structure, thus forming the helix-turn-helix (HTH) fold. Based on the type of conserved MYB domain, the *Os2R-MYB* proteins were classified into five subgroups (Figure 2). This conserved tryptophan (W), regularly repeated, is essential for maintaining the hydrophobic function of proteins. The first W of the R3 is usually replaced by aromatic or hydrophobic amino acids, such as leucine (L), isoleucine (I), or phenylalanine (F) [2,3,27]. In the *Os2R_MYB* protein of Group_2B, the first W of the R3 domain was replaced by F. In Group_2C, all of the first W of the R3 domain were replaced by F except for Os2R_MYB95/68/67/17, while most of the W were replaced by F in Group_2D, Group_2E, and Group_2F. In a few cases, the W was replaced by I or L. Interestingly, the R2 and R3 had swapped places in Group_2A.

### 2.3. Structure of Os2R_MYBs Genes

**Figure 3:**
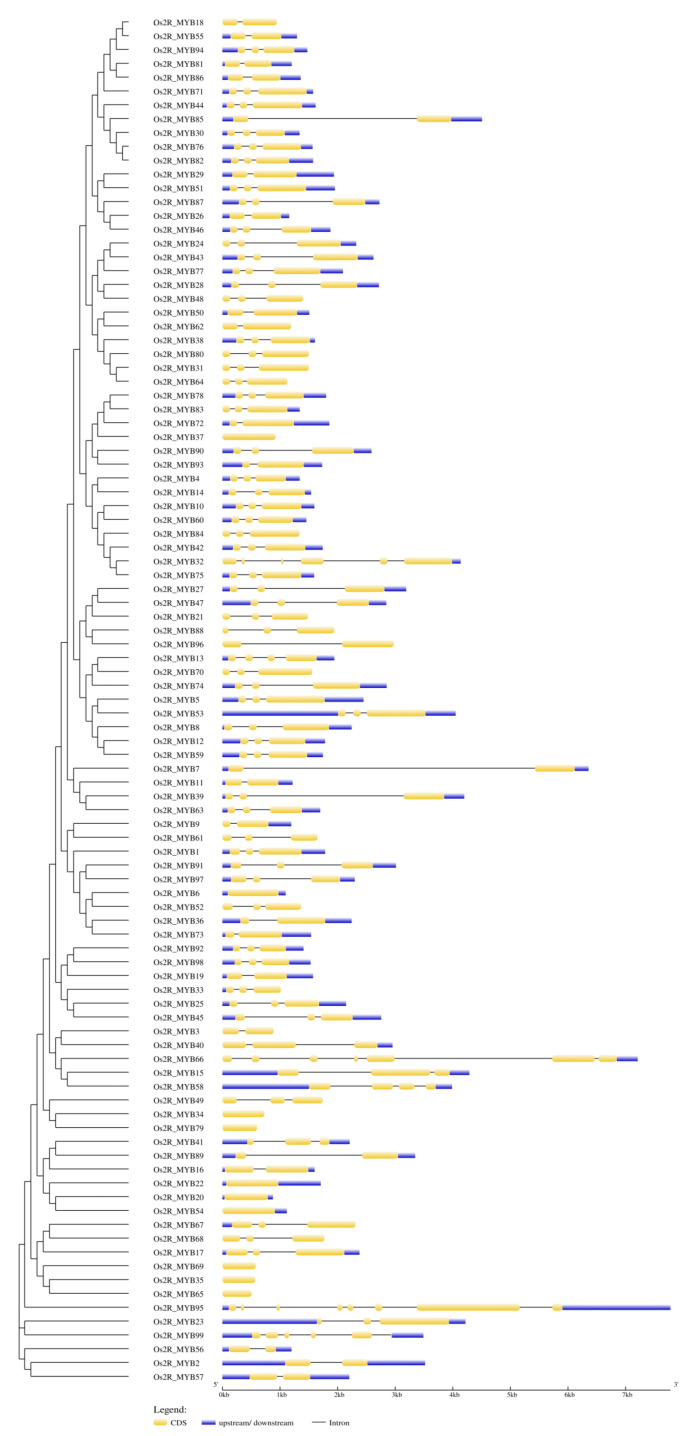
Phylogenetic tree and intron/exon structure of *Os2R_MYB* genes. The blue boxes represent the 5′ or 3′ untranslated regions (UTR), the yellow boxes represent the coding sequences (CDS), and the black lines represent the intron regions.

**Figure 4:**
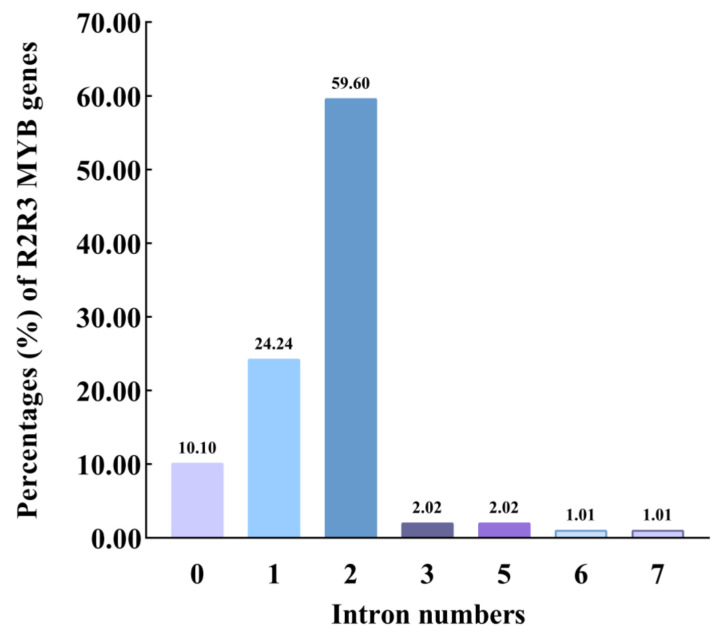
Statistical analysis of the intron numbers of 99 *Os2R_MYB* genes. The bar chart shows the proportion of *Os2R_MYB* genes with different intron numbers in the 99 *Os2R_MYB* genes.

Exon and intron distribution profiles of *Os2R_MYBs* were analyzed by GSDS V2.0 to align corresponding genomic sequences (Figure 3), and the number of introns was statistically analyzed (Figure 4). The result showed that most *Os2R_MYB* genes contained two introns (59, 59.6%). *Os2R_MYB66* contained six introns, and *Os2R_MYB95* contained seven introns, while ten genes were intronless (Table S2).

### 2.4. Analysis of Promoter Cis-Elements

**Figure 5:**
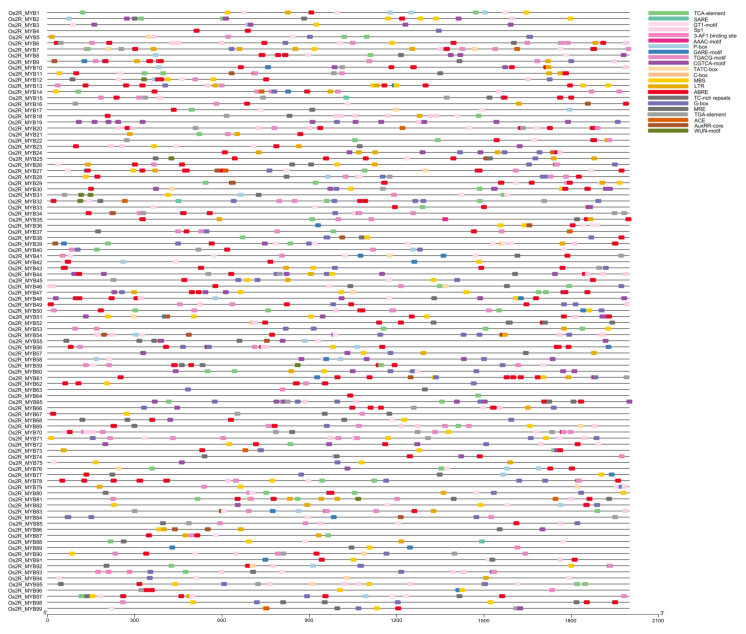
Distribution of cis-elements in promoter sequences of 99 *Os2R_MYB* genes. TCA-element/SARE, cis-acting element involved in salicylic acid responsiveness; MBS, MYB binding site involved in drought-inducibility; GT1-motif/Sp1/3-AF1/AAAC, light-responsive element; LTR, cis-acting element involved in low-temperature responsiveness; ABRE, cis-acting element involved in the abscisic acid responsiveness; P-box/GARE, gibberellin-responsive element; TGACG/CGTCA motifs, involved in the MeJA responsiveness; TC-rich repeats, cis-acting element involved in defense and stress responsiveness; MRE, MYB binding site involved in light responsiveness; TATC-box/C-box, cis-acting element involved in gibberellin responsiveness; TGA-element, auxin-responsive element; G-box/ACE, cis-acting element involved in light responsiveness; AuxRR-core, involved in auxin responsiveness; WUN-motif, wound-responsive element.

**Figure 6:**
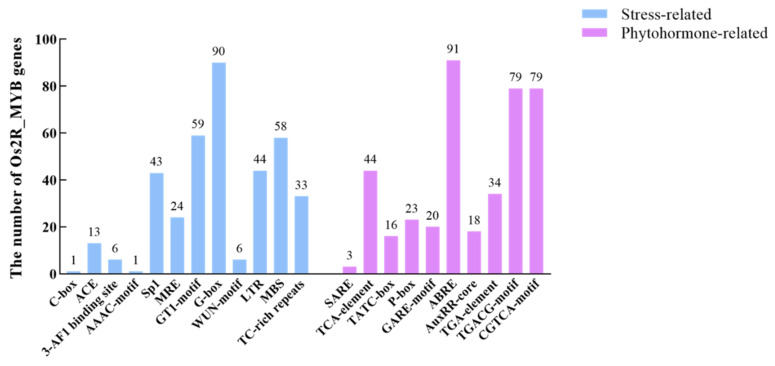
Statistical analysis of the cis-elements in 2000 bp regions upstream of *Os2R_MYB* genes. The bar chart shows the number of *Os2R_MYB* genes with various cis-acting elements in 99 *Os2R_MYB* genes. The light blue and purple bars represent stressed-related and phytohormone-related cis-acting elements, respectively.

To further understand the regulatory mechanism of the *Os2R_MYB* genes in plant growth, development, and stress response, the 2000 bp regions upstream of 99 *Os2R_MYBs* were analyzed. 10 hormone and 12 stress-response cis-elements (Figure 5 and Figure 6) were detected in the promoter region of the *Os2R_MYBs*. Ten hormone-responsive cis-elements included the TCA-Element/SARE (salicylic acid response), the ABRE (abscisic acid response), the TATC-box/P-box/gare-motif (gibberellin response), the TGACG-motif/CGTCA-motif (MeJA response), and the TGA-element/AuxRR-core (auxin response). Twelve stress-related elements included the GT1-motif/G-box/MRE/Sp1/ACE/3-AF1 binding site/AAAC-motif (light), the MBS (drought), the LTR (low temperature), TC-rich repeats (defense and stress response), and the WUN-motif (wound). A total of 91 *Os2R_MYB* genes had the ABRE motif, indicating the potential responses to ABA. The TGACG or the CGTCA motifs were detected in 79 *Os2R_MYB* genes, indicating that they were sensitive to methyl jasmonic acid. Fifty-eight *Os2R_MYB* genes had the MBS motif, indicating that they were involved in the drought-stress response of plants (Table 1).

### 2.5. The Relative Expression Level of Os2R_MYBs in Various Tissues and Growth Stages

**Figure 7:**
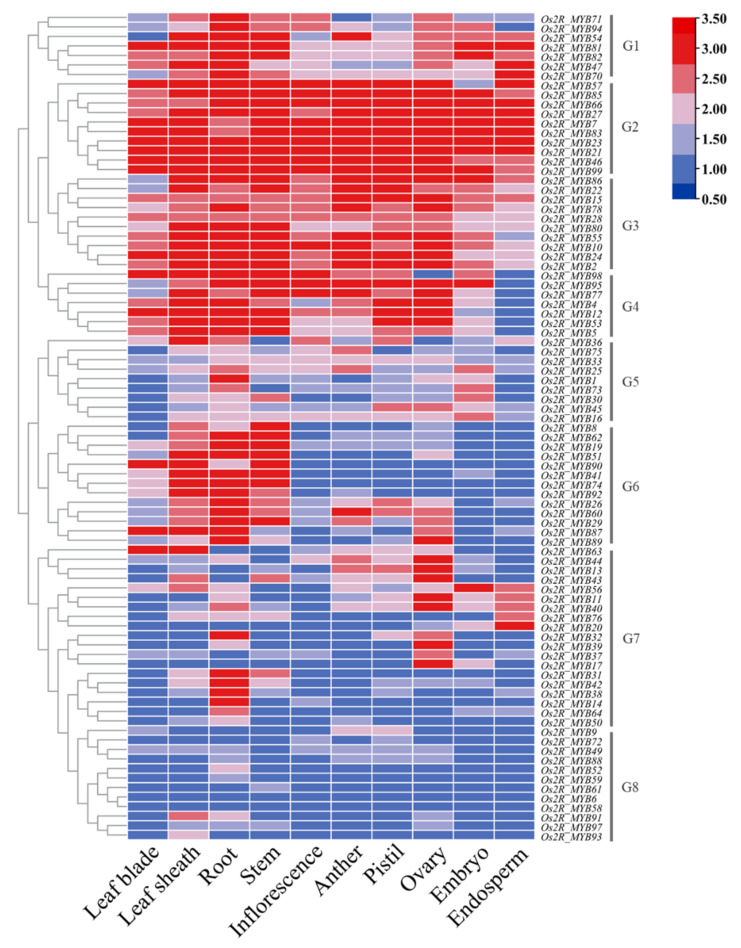
The relative expression level of *Os2R_MYB* genes in various tissues and growth stages. Lower expression is represented with dark blue; higher expression is represented with red. Leaf-blade, vegetative leaf-blade; Leaf-sheath, vegetative leaf-sheath; Root, vegetative root; Stem, reproductive stem; Inflorescence, 0.6–1 mm; Anther, 0.7–1 mm; Pistil, 5–10 cm; Ovary, 3 DAF; Embryo, 10 DAF; Endosperm, 42 DAF.

To explore the function of *Os2R_MYBs*, we dissected the expression levels of *Os2R_MYB* genes in various tissues and growth stages (Figure 7). Based on the expression analysis, these genes were classified into eight groups (G1-G8) according to cluster analysis results. Group G1 contained 7 *Os2R_MYB* genes, all highly expressed in roots, implying that these genes might be involved in root development. Most genes in G2 and G3 groups were highly expressed in all tissues, indicating their universal roles. Seven *Os2R_MYB* genes in the G4 group were all lowly expressed in the endosperm, implying that these genes may not be involved in endosperm development. The highly expressed MYB genes were mainly located in leaf sheath, root, and stem but showed low expression in other tissues or organs in G6. The expression level of G8 genes was lower in all tissues.

### 2.6. Protein–Protein Interaction Network Analysis

**Figure 8:**
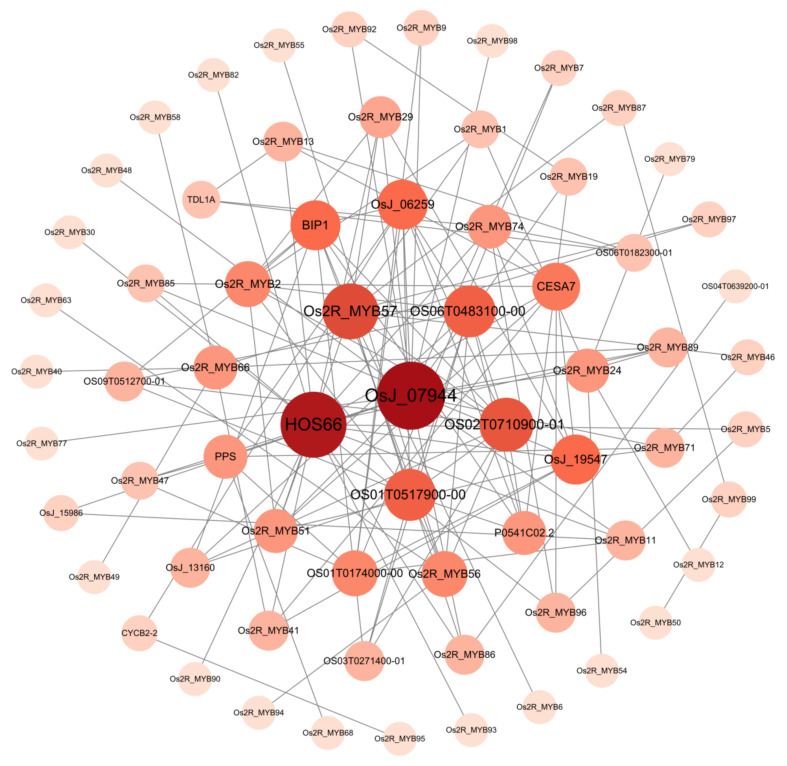
Interaction analysis of R2R3_MYB proteins using the STRING servers in rice. The nodes represent different proteins, and the edges represent the interaction between proteins. Proteins are arranged according to the degree (degree = 1–18) algorithm in Cytoscape. The value of degree corresponds to the color shade and size of nodes.

Various proteins interact with each other to form a protein interaction network, which helps to regulate the biological signal transmission and related gene expression. In order to further study the function of R2R3_MYB proteins in rice, we analyzed the protein-protein interaction network of 99 *Os2R_MYBs* (Figure 8 and Table S3). Protein–protein interaction network protein annotations and functional annotations are shown in Tables S4 and S5. The result showed that OsJ_07944, HOS66, Os2R_MYB57, OS01T0517900-00, and OS02T0710900-01 played pivotal roles in this network and were involved in metabolic process, biosynthetic process, and tissue development in plant. There was a putative interaction between Os2R_MYB57 and Os2R_MYB1/6/29/46/51/87/97, BIP1, and OS02T0710900-01, in which BIP1 was involved in rice endosperm regulation, suggesting that Os2R_MYB57 may have a similar function [28]. The OsJ07944 (OS02T0682500-01), a putative anthocyanin biosynthetic gene regulator PAC1, interacted with Os2R_MYB11/19/24/29/47/48/51/71/77/92/98/89.

### 2.7. Gene Expression Levels of Os2R_MYBs under Abiotic Stresses

**Figure 9:**
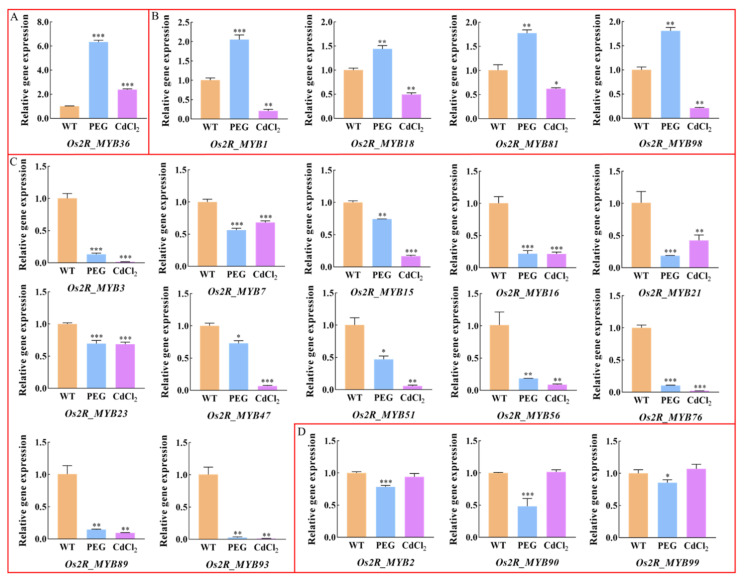
Relative gene expression of 20 *Os2R_MYBs* under PEG and CdCl_2_ stress treatments. (**A**–**D**) represent different expression patterns under PEG and CdCl_2_ stress treatment. Nipponbare cultivar was used as the control; *OsActin1* was used as a reference gene. The relative expression levels of each gene were calculated by the 2^−ΔΔCt^ method. All reactions were set up in triplicate. Bars represent the standard deviation (SD) of the three replicates. A significant difference is indicated by an asterisk according to *t*-test (* *p* < 0.05, ** *p* < 0.01, and *** *p* < 0.001).

We selected 20 *Os2R_MYB* genes and analyzed their expression levels under either PEG or CdCl_2_ treatments by qRT-PCR (Figure 9 and Figure S2). The results indicated that most of the 20 *Os2R_MYB* genes exhibited altered expression upon PEG or CdCl_2_ stress induction. Five *Os2R_MYB* genes were upregulated during PEG treatment. The expression levels of *Os2R_MYB36, Os2R_MYB1, Os2R_MYB18, Os2R_MYB81,* and *Os2R_MYB98* were significantly upregulated. The gene expression levels of twelve *Os2R_MYBs*, namely *Os2R_MYB3, Os2R_MYB7, Os2R_MYB15, Os2R_MYB16, Os2R_MYB21, Os2R_MYB23, Os2R_MYB56, Os2R_MYB76, Os2R_MYB89, Os2R_MYB93, Os2R_MYB2,* and *Os2R_MYB99*, were significantly downregulated. Under CdCl_2_ stress treatment, the expression level of *Os2R_MYB36* was significantly upregulated. *Os2R_MYB3, Os2R_MYB7, Os2R_MYB15, Os2R_MYB23, Os2R_MYB47, Os2R_MYB76, Os2R_MYB89*, and *Os2R_MYB93* genes were significantly downregulated. However, the expression level of *Os2R_MYB2, Os2R_MYB90,* and *Os2R_MYB99* genes had no obvious alteration. In general, the twelve genes’ (*Os2R_MYB3/7/15/16/21/23/47/51/56/76/89/93*) expression levels were downregulated, only one gene (*Os2R_MYB36*) was upregulated, and four genes (*Os2R_MYB1/18/81/98*) had absolutely opposite expression patterns under drought and cadmium stresses. The statistical results are shown in Table S6. Among these genes, *Os2R_MYB1* is involved in phosphorus starvation signaling and gibberellin as a regulatory factor [29]. *Os2R_MYB90* regulates phosphorus accumulation and phosphorus-responsive transporters in rice, and overexpression of *Os2R_MYB90* enhances plant growth and root system architecture [30]. The *Os2R_MYB36*-overexpressing plants were more sensitive to abscisic acid and more tolerant to cold, dehydration, and salt stresses than control plants [31]. These results showed that the selected *Os2R_MYB* genes were responsive to abiotic stress, suggesting a potential role involved in the stress regulation.

## 3. Discussion

MYB TF family is one of the most abundant families in plants, with complex and diverse functions. This study identified 190 MYB family members in the rice genome unevenly distributed on all 12 chromosomes (Figure 1), of which 99 were R2R3-MYB (52.1%). R2R3-MYB group is one of the largest group of proteins in plants. They all contain two R structures at the N-terminal but are variable at the C-terminal containing transcriptional activation or inhibition domains, and conserved threonine and serine residues served as post-translational modification sites [5,32]. R2R3-MYB TFs are extensively involved in hormone response, cell differentiation, secondary metabolism, abiotic stress, resistance to disease, and insect infestation [33,34,35,36,37]. For example, overexpression of *SG3*, an R2R3-MYB protein-encoding gene, can promote rice grain elongation [38]. The R2R3-MYB transcription factor MYB106 in *Arabidopsis thaliana* is a negative regulator of plant flowering. In the mutant without *MYB106*, plants show early flowering in long-day growth condition [39]. Overexpression of *GmMYB81* endows plants with tolerance to salt and drought stress [36]. The “-W-(X18/X19)-W-(X18/X19)-W-…W/F/I/L-W(X18/X19)-W-” structure in the N-terminal of MYB domain is the key sequence of its complex functions. We aligned the sequences of the 2R-MYB protein of rice (Figure S1), and the results showed that almost all 99 2R-MYB proteins contained the structure. The first W of the R3 domain was usually replaced by aromatic or hydrophobic amino acids such as leucine (L), isoleucine (I), or phenylalanine (F), which together form a hydrophobic core [2,27], conferring their complex functions. Interestingly, the R2 and R3 had swapped places in Os2R_MYB2/56/57. We constructed phylogenetic trees of MYB proteins in *Oryza sativa* L. and *Arabidopsis thaliana* (Figure 2) and classified them into five subgroups based on the key sequence features. Most plant proteins are hydrophilic, and thus, some protein partners or signal peptides are needed to participate the transportation and localization of proteins through lipid bilayer. For example, in soybean, previous studies have shown that thylakoid transport peptides affect the expression and localization of NdhB subunits [40]. Analysis of the physicochemical properties of *Os2R_MYB* protein (Table S1) showed that most types of 2R-MYB proteins were hydrophilic, and a similar functional mechanism might exist, requiring further study.

Gene structure is important to the study of gene evolution. To better comprehend the evolutionary relationship of the *Os2R_MYB* genes, their exon and intron distribution profiles were analyzed and counted (Figure 3 and Figure 4). The result showed that most *Os2R_MYB* genes were evolutionarily conserved and contained two introns (59, 59.6%). *Os2R_MYB66* contained six introns, and *Os2R_MYB95* contained seven introns, while ten genes were intronless (Table S2).

In this study, we used the predicted gene expression data to dissect the expression patterns of *Os2R_MYB* genes in various tissues and growth stages (Figure 7), and combined with the 2R-MYB protein phylogenetic tree cluster analysis in rice and Arabidopsis (Figure 2), the functions of some *Os2R_MYB* genes could be predicted. At2R_MYB52 (At3g27810) and At2R_MYB105 (At5g40350) are both induced by jasmonate and have an important effect in stamen development [41]. At2R_MYB112 (At5g56110) is clustered with Os2R_MYB44 (Os04g0470600), *Os2R_MYB44* is a key regulator of tapetal development and pollen wall formation, and *At2R_MYB112* is also involved in the regulation of pollen wall formation [42,43]. These results suggested the reliability of our phylogenetic tree clustering. In addition, At2R_MYB33 (At2g32460) and Os2R_MYB58 (Os05g0490600) are clustered, and *At2R_MYB33* may mediate gibberellin signaling in flowering responses, conferring their functions in root tip and subsequent stem tissue during germination [44]. *Os2R_MYB66* was highly expressed in roots, stems, and inflorescences, suggesting that *Os2R_MYB66* might have similar functions to *At2R_MYB33*.

Protein–protein network analysis shows that the *Os2R_MYB* protein families play a significant role in biological signal transmission and gene expression regulation of the life process (Figure 8). The mechanism of action of miRNA is directly related to protein, and miRNA plays its biological function mainly by participating in the regulation of downstream gene translation process. Abnormal protein expression is closely related to the development and stress resistance of plants. Further study of the miRNA interaction network will help provide a basis for future functional analysis. For example, previous studies have clarified the functions of *TaTALEs* in development and stress response through miRNA interaction network analysis [45,46]. We further explored *Os2R_MYB* genes in the abiotic stress response and found that *Os2R_MYB36* gene expression levels were significantly higher, while *Os2R_MYB3/7/15/16/21/23/47/51/56/76/89/93* gene expression levels were significantly lower (Figure 9, Figure S2 and Table S6) under the stress of drought and cadmium. These results exhibited that these genes were involved in regulating rice anti-stress response. Except for *Os2R_MYB89*, ABRE elements were existent in the promoter regions of these genes (Figure 5 and Figure 6), suggesting that these genes may be involved in the response to drought and heavy metal stress via ABA signaling pathway. The promoter regions of *Os2R_MYB18/81/98/23* all contain TC-rich repeats, indicating that these genes are involved in plant defense response and environmental stress. In plants, the salicylic acid (SA) is closely related to the formation of plasmodesmata, promoting bud germination [47,48]. The promoter region of *Os2R_MYB15/32/88* contained SARE, indicating that these genes may be involved in the regulation of SA, which is involved in plant growth and development.

## 4. Materials and Methods

### 4.1. Identification of MYB Family Members in Rice and Their Chromosomal Locations

The DNA and amino acid sequences of MYB TF of rice were downloaded from the MSU database (http://rice.uga.edu/index.shtml) (accessed on 11 November 2021) [49] based on the pfam family number (PF00249), generating a list of 427 DNA and protein sequences. Redundant sequences were deleted through the CD-Hit website (http://weizhong-lab.ucsd.edu/cdhit-web-server/cgi-bin/index.cgi?cmd=cd-hit) (accessed on 11 November 2021) [50], and 239 non-redundant protein sequences were retained. Then, the non-redundant protein sequences were entered in the Pfam website (http://pfam.xfam.org/search#tabview=tab1) (accessed on 11 November 2021) for domain confirmation. The non-typical MYB domains and short sequences were removed manually, delivering a list of 190 MYB proteins that were identified and renamed finally using the TBtools. The distribution of the *MYB* gene on the 12 chromosomes of rice was visualized by TBtools based on chromosome location information. The MYB protein list derived from the MSU database was loaded into ExPASy (https://web.expasy.org/protparam/?tdsourcetag=s_pcqq_aiomsg) (accessed on 11 November 2021) [51] to obtain the respective physicochemical properties, including molecular weight (MV), numbers of amino acids (N), isoelectric point (pI), grand average of hydropathicity (GRAVY), and instability index.

### 4.2. Classification and Phylogenetic Analysis of MYB Family Members

The Arabidopsis MYB transcription factors were downloaded from TAIR (https://www.arabidopsis.org/tools/index.jsp) (accessed on 11 August 2021). Clustal Omega [52] was used to align the MYB proteins derived from Arabidopsis and rice. The MYB protein family phylogenetic tree was constructed by the neighbor-joining method of Mega-X [53] with a bootstrap value set to 1000. Subsequently, the generated dendrogram was polished by the Evolview (https://evolgenius.info//evolview-v2/) (accessed on 13 July 2022).

### 4.3. Structural Analysis of Os2R_MYB Genes

To identify the introns and exons of the rice 2R-MYB gene sequences, the GSDS platform (http://gsds.gao-lab.org/) (accessed on 13 August 2021) [54] was used and were further visualized with TBtools.

### 4.4. Analysis of Stress-Related Cis-Elements in Os2R_MYB Promoter Regions

The 2000 bp DNA sequences upstream of the 2R-MYB TF corresponding to their promoters were extracted from the RAP-DB database (https://rapdb.dna.affrc.go.jp/tools/dump) (accessed on 18 November 2021) [49,55]. Stress response cis-elements in promoter sequences were analyzed using the PlantCARE database (http://bioinformatics.psb.ugent.be/webtools/plantcare/html/) (accessed on 18 November 2021) [56].

### 4.5. Analysis of the Relative Expression Level of Os2R_MYBs in Different Tissues and Growth Stages

The expression data of the rice 2R-MYB genes from various tissues and growth stages were downloaded from RiceXPro (https://ricexpro.dna.affrc.go.jp/GGEP/index.php) (accessed on 25 November 2021). A total of 10 samples were collected, including leaf blades, leaf sheaths, roots, stems, inflorescence, anthers, pistils, ovaries, embryos, and endosperm. The profiles of *Os2R_MYB* genes were plotted by TBtools and normalized by lgN, and the expression levels were presented with different colors.

### 4.6. Abiotic Stress Treatment of Rice

Nipponbare cultivar was used as the experimental material. Rice seeds were disinfected with 2.5% NaClO and washed with sterile water. After three days on a wet filter paper at 37 °C, the rooted seeds were transferred to hydroponic boxes. At the two-leaf stage, the seedlings were treated either with 20% 6000 PEG or 50 μmol/L CdCl_2_ for 24 h to simulate the growth of rice seedlings under environmental stress such as drought and cadmium. Rice leaves after stress treatments were collected and stored at −80 °C for RNA extraction.

### 4.7. Protein–Protein Interaction Network Analysis

The 2R-MYB-type protein list derived from the MSU database was loaded into STRING online website (https://string-db.org/cgi/input?sessionId=byDrTWHDQ5tp&input_page_active_form=multiple_identifiers) (accessed on 15 July 2022) to predict putative interacting proteins [57]. The protein–protein interaction network was generated and decorated by Cytoscape software.

### 4.8. Analysis of Os2R_MYBs Gene Expression in Rice under Abiotic Stress

Based on the observed variation in gene expression, 20 MYB genes were selected to detect the changes in transcription levels under the selected stress inducers by qRT-PCR. The primers were designed by Beacon Designer 7.9, and the sequence information of all primers is shown in Table S7. Total RNA from rice leaves was extracted by Easy Plant RNA Extraction Kit, and the first-strand cDNA was synthesized using FastKing RT Kit. Then, cDNA samples were diluted to 200 ng/μL to serve as templates for qRT-PCR. The reaction was carried out using UltraSYBR Green Master Mixture in a Bio-Rad CFX system. Reaction conditions: 4 min at 95 °C, followed by 39 cycles of 15 s at 95 °C, 15 s at 58 °C, and 25 s at 72 °C. The cultivar Nipponbare was used as the control, while the rice *Actin1* and *GAPDH* were used as reference genes. The relative expression levels of each gene were calculated by the 2^−ΔΔCt^ method. All reactions were set up in triplicates. Standard deviation (SD) was evaluated from three replicates.

## 5. Conclusions

A total of 190 MYB proteins were identified from rice genome data, including 99 2R-MYB-type proteins. The genes encoding these proteins were unevenly distributed across 12 chromosomes of rice. There were 10 duplicate gene pairs. *Os2R_MYB* protein was classified into five subgroups according to its sequence characteristics, and the sequence characteristics of these proteins were further verified by multiple sequence alignment. We also dissected the structure of the genes encoding these proteins, and the result showed that most *Os2R_MYB* genes contained two introns (59, 59.6%). Moreover, their cis-elements in the upstream 2000 bp promoter region were analyzed, suggesting their involvement in rice hormone response or abiotic stress response. The *Os2R_MYB* proteins may participate in biological signal transmission and gene expression regulation by protein–protein interaction network analysis. Subsequent studies can further clarify the function of the *Os2R_MYB* protein by studying a specific *Os2R_MYB* protein–protein interaction proteins and co-expression genes. The differential expression of 20 *Os2R_MYB* genes under 6000PEG and CdCl_2_ treatments showed that some *Os2R_MYB* genes responded to PEG and/or CdCl_2_ stresses. This study can serve as a basis for the identification and functional study of the MYB TF family members of rice and help to illustrate the molecular mechanism of rice response to abiotic stress, which is expected to breed new rice varieties with strong resistance to abiotic stress.

## Figures and Tables

**Table 1:** Statistical analysis of the cis-elements in 2000 bp regions upstream of *Os2R_MYB* genes.

Type	Cis-Elements	Gene Numbers	Percentages
Stress-related	C-box	1	1.0%
	ACE	13	13.1%
	3-AF1 binding site	6	6.1%
	AAAC-motif	1	1.0%
	Sp1	43	43.4%
	MRE	24	24.2%
	GT1-motif	59	59.6%
	G-box	90	90.9%
	WUN-motif	6	6.1%
	LTR	44	44.4%
	MBS	58	58.6%
	TC-rich repeats	33	33.3%
Phytohormone-related	SARE	3	3.0%
	TCA-element	44	44.4%
	TATC-box	16	16.2%
	P-box	23	23.2%
	GARE-motif	20	20.2%
	ABRE	91	91.9%
	AuxRR-core	18	18.2%
	TGA-element	34	34.3%
	TGACG-motif	79	79.8%
	CGTCA-motif	79	79.8%

## Data Availability

Not applicable.

## Supplementary Materials

The following supporting information can be downloaded at: https://www.mdpi.com/article/10.3390/plants11151928/s1, Figure S1: Multiple sequence alignments of Os2R_MYB proteins; Figure S2: Relative gene expression of 20 Os2R_MYBs under PEG and CdCl_2_ stress treatments; Table S1: Physicochemical Properties of the 2R-MYB Type Proteins; Table S2: Statistical Analysis of the Intron Numbers of 99 Os2R_MYB Genes; Table S3: R2R3_MYB proteins interaction network construct; Table S4: R2R3_MYB proteins interaction network protein annotations; Table S5: R2R3_MYB proteins interaction network functional annotations; Table S6: Statistical Analysis of Os2R_MYB Genes Expression Level under the PEG and CdCl2 Stresses; Table S7: Quantitative RT-PCR Primers.
