# Supplementary material for: Genome-Wide Identification of R2R3-MYB Transcription Factor and Expression Analysis under Abiotic Stress in Rice
Source: Plants (Basel). 2022 Jul 25;11(15):1928. doi: 10.3390/plants11151928 (PMC9330779; doi:10.3390/plants11151928)
Supplement: Supplementary file 1 [file plants-11-01928-s001.zip › Figure S1 Multiple sequence alignments of Os2R_MYB proteins.pdf]

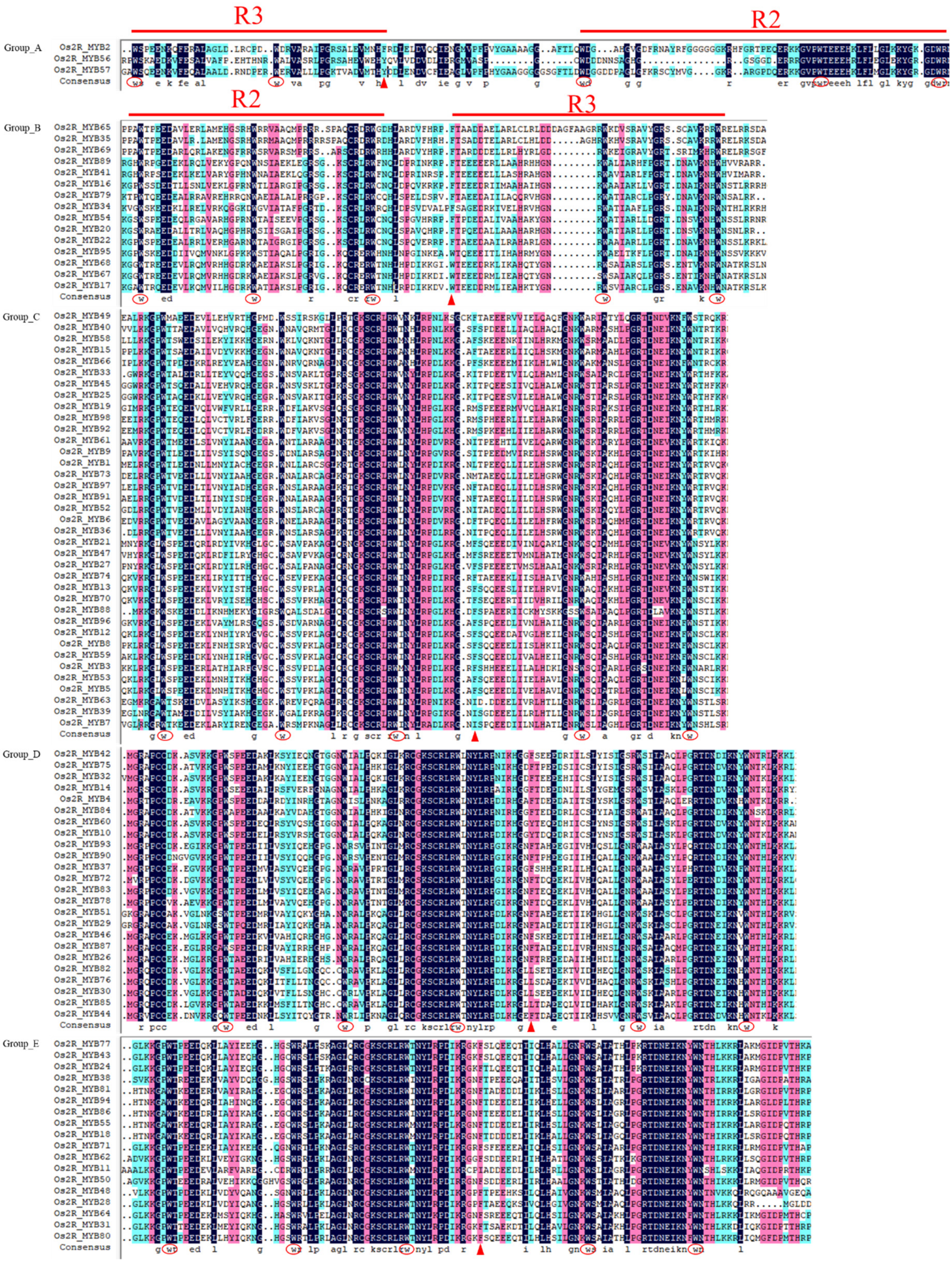

Figure S1. Multiple sequence alignments of Os2R_MYB proteins. The conserved tryptophan (W) of the "-W-(X18/X19)-W-(X18/X19)-W-…-W(X18/X19)-W-" structure has been marked with red circle. The red triangle represents W replaced by F/I (isoleucine) /L (leucine) /Y (tyrosine). Black highlight represents a homology level of 100%, pink ≥ 75%, and blue ≥ 50%.
