# Supplementary material for: Genome-Wide Identification of R2R3-MYB Transcription Factor and Expression Analysis under Abiotic Stress in Rice
Source: Plants (Basel). 2022 Jul 25;11(15):1928. doi: 10.3390/plants11151928 (PMC9330779; doi:10.3390/plants11151928)
Supplement: Supplementary file 1 [file plants-11-01928-s001.zip › Figure S2 Relative gene expression of 20 Os2R_MYBs under PEG and CdCl2 stress treatments.pdf]

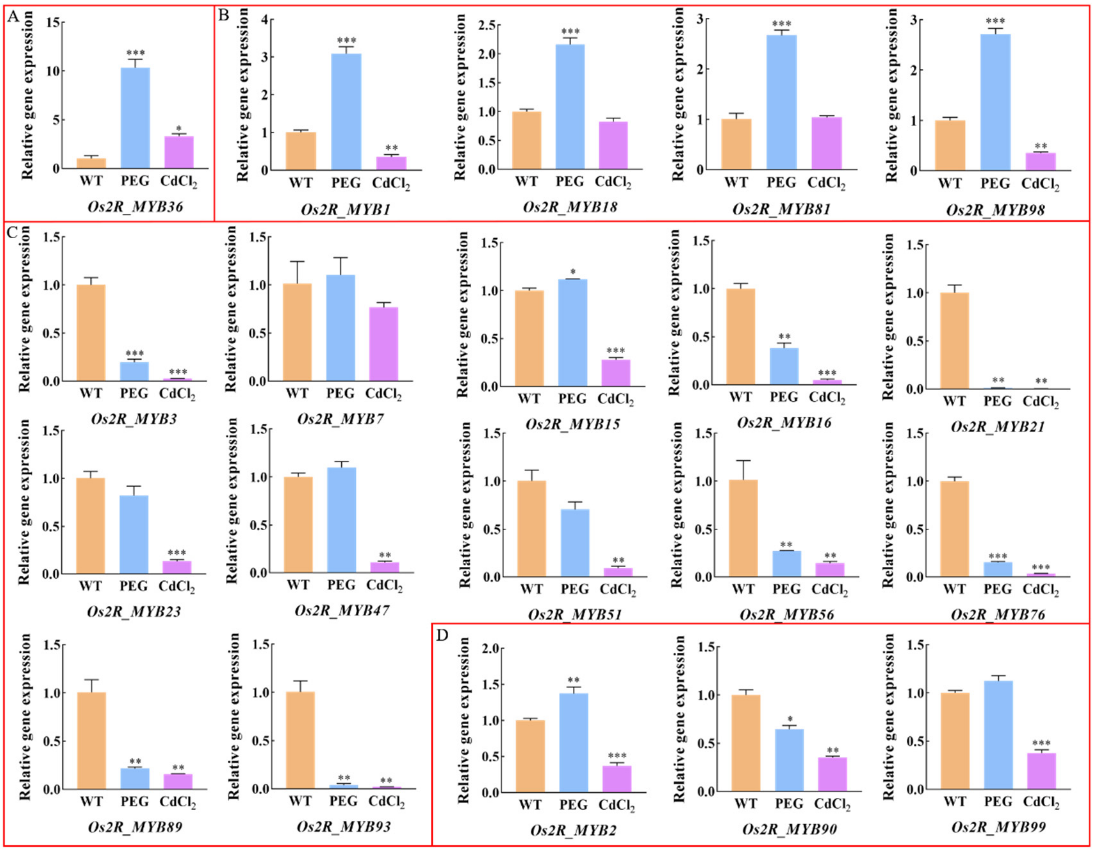

Figure S2. Relative gene expression of 20 *Os2R_MYBs* under PEG and $CdCl_2$ stress treatments. A, B, C and D represent different expression patterns under PEG and $CdCl_2$ stress treatment. Nipponbare cultivar was used as the control, *OsGAPDH* was used as a reference gene. The relative expression levels of each gene were calculated by the $2^{-\Delta\Delta Ct}$ method. All reactions were set-up in triplicates. Bars represent the standard deviation (SD) of the three replicates. A significant difference is indicated by an asterisk according to t-test (* $p < 0.05$, ** $p < 0.01$, and *** $p < 0.001$).
