# Supplementary material for: Genome-Wide Identification of R2R3-MYB Transcription Factor and Expression Analysis under Abiotic Stress in Rice
Source: Plants (Basel). 2022 Jul 25;11(15):1928. doi: 10.3390/plants11151928 (PMC9330779; doi:10.3390/plants11151928)
Supplement: Supplementary file 1 [file plants-11-01928-s001.zip › Table S1 Physicochemical Properties of the 2R-MYB Type Proteins.pdf]

Table S1. Physicochemical Properties of the 2R-MYB Type Proteins

Name	Number of amino acids	Molecular weight	Theoretical pI	Instability index	Grand average of hydropathicity (GRAVY)
Os2R_MYB1	350	38488.30	6.07	58.76	-0.486
Os2R_MYB2	294	32501.60	9.40	55.91	-0.584
Os2R_MYB3	260	27583.70	8.85	56.56	-0.521
Os2R_MYB4	264	27979.34	5.73	60.22	-0.286
Os2R_MYB5	428	47736.41	6.20	62.79	-0.671
Os2R_MYB6	293	32507.01	5.68	56.09	-0.589
Os2R_MYB7	317	33646.14	5.50	49.04	-0.732
Os2R_MYB8	357	38499.69	5.70	61.29	-0.590
Os2R_MYB9	228	25448.51	6.32	52.77	-0.552
Os2R_MYB10	318	33868.21	8.27	63.41	-0.363
Os2R_MYB11	275	29793.33	5.80	47.45	-0.543
Os2R_MYB12	301	33199.94	5.81	53.22	-0.543
Os2R_MYB13	307	32777.98	8.07	50.38	-0.226
Os2R_MYB14	298	31761.19	4.88	60.41	-0.427
Os2R_MYB15	553	59960.66	5.08	48.96	-0.508
Os2R_MYB16	413	44329.57	6.24	64.04	-0.575
Os2R_MYB17	448	48075.22	7.75	58.95	-0.439
Os2R_MYB18	287	30969.95	9.30	61.61	-0.572
Os2R_MYB19	276	31029.37	6.86	69.20	-0.837
Os2R_MYB20	250	27339.30	9.91	61.76	-0.439
Os2R_MYB21	297	32118.73	7.80	59.03	-0.602
Os2R_MYB22	301	32322.17	8.85	72.59	-0.630
Os2R_MYB23	470	51199.37	8.50	55.69	-0.677
Os2R_MYB24	342	36317.56	6.60	47.78	-0.395
Os2R_MYB25	289	32082.79	5.35	55.35	-0.679
Os2R_MYB26	258	28468.84	5.92	60.17	-0.618
Os2R_MYB27	317	34205.52	5.25	68.89	-0.731
Os2R_MYB28	300	32154.28	6.96	42.16	-0.353
Os2R_MYB29	340	36443.44	4.67	43.95	-0.580
Os2R_MYB30	255	27759.31	5.20	50.01	-0.471
Os2R_MYB31	377	40296.57	6.25	58.20	-0.352
Os2R_MYB32	563	58761.40	5.59	52.68	-0.410
Os2R_MYB33	247	28768.39	6.60	72.96	-0.879
Os2R_MYB34	242	25320.00	9.82	57.06	-0.305
Os2R_MYB35	189	22195.49	11.36	89.07	-0.644
Os2R_MYB36	329	36042.85	4.84	59.44	-0.613
Os2R_MYB37	307	32479.70	6.59	55.58	-0.262
Os2R_MYB38	317	33199.38	8.17	51.34	-0.269
Os2R_MYB39	321	34048.96	6.16	54.02	-0.543
Os2R_MYB40	522	55458.72	5.13	65.83	-0.476
Os2R_MYB41	245	27525.51	8.22	57.26	-0.906
Os2R_MYB42	321	35339.26	7.61	55.53	-0.486

Name	Number of amino acids	Molecular weight	Theoretical pI	Instability index	Grand average of hydropathicity (GRAVY)
Os2R_MYB43	345	36392.82	7.24	46.94	-0.417
Os2R_MYB44	372	39699.24	5.77	40.31	-0.537
Os2R_MYB45	281	30659.25	7.09	67.63	-0.640
Os2R_MYB46	257	27914.37	6.56	56.90	-0.591
Os2R_MYB47	273	29536.02	6.07	54.84	-0.514
Os2R_MYB48	298	31799.50	6.00	38.87	-0.365
Os2R_MYB49	343	37110.93	7.69	70.61	-0.543
Os2R_MYB50	340	36876.57	6.20	52.17	-0.363
Os2R_MYB51	371	39768.54	4.94	56.25	-0.518
Os2R_MYB52	310	33960.04	5.65	66.07	-0.388
Os2R_MYB53	427	47151.65	6.12	61.20	-0.656
Os2R_MYB54	303	31528.14	6.61	59.62	-0.450
Os2R_MYB55	260	28351.23	9.06	51.43	-0.608
Os2R_MYB56	182	20381.59	7.11	62.40	-0.723
Os2R_MYB57	315	34031.20	6.49	60.09	-0.533
Os2R_MYB58	394	45133.28	5.19	47.06	-0.786
Os2R_MYB59	312	33606.16	5.18	47.51	-0.395
Os2R_MYB60	289	30551.35	6.09	60.18	-0.299
Os2R_MYB61	249	26862.71	5.97	45.85	-0.492
Os2R_MYB62	367	39053.13	5.62	50.32	-0.387
Os2R_MYB63	271	28895.07	6.82	47.41	-0.469
Os2R_MYB64	322	36126.04	5.82	53.75	-0.468
Os2R_MYB65	169	19018.54	10.77	91.68	-0.590
Os2R_MYB66	682	75878.94	7.31	54.68	-0.771
Os2R_MYB67	438	47467.99	5.71	54.42	-0.544
Os2R_MYB68	329	35943.62	8.25	56.95	-0.603
Os2R_MYB69	192	22123.39	10.40	82.19	-0.665
Os2R_MYB70	400	41655.86	6.40	49.84	-0.237
Os2R_MYB71	368	39929.04	6.59	55.13	-0.408
Os2R_MYB72	341	35258.90	5.90	47.08	-0.498
Os2R_MYB73	300	33702.95	5.52	59.14	-0.502
Os2R_MYB74	359	39954.66	6.13	50.48	-0.647
Os2R_MYB75	316	34397.20	5.89	62.48	-0.644
Os2R_MYB76	309	34282.98	5.41	60.33	-0.511
Os2R_MYB77	357	39826.53	6.15	59.04	-0.732
Os2R_MYB78	310	32785.77	7.69	50.15	-0.592
Os2R_MYB79	200	21605.32	6.12	65.11	-0.458
Os2R_MYB80	357	36754.20	6.10	47.90	-0.178
Os2R_MYB81	243	26999.11	9.10	55.43	-0.554
Os2R_MYB82	279	30935.94	5.03	54.69	-0.506
Os2R_MYB83	321	33068.81	8.17	48.20	-0.540
Os2R_MYB84	377	40137.68	6.06	53.99	-0.427
Os2R_MYB85	285	31171.89	5.83	53.16	-0.570

Name	Number of amino acids	Molecular weight	Theoretical pI	Instability index	Grand average of hydropathicity (GRAVY)
Os2R_MYB86	251	28224.49	9.04	48.25	-0.543
Os2R_MYB87	276	30064.68	7.71	59.19	-0.651
Os2R_MYB88	294	31656.64	5.22	68.39	-0.411
Os2R_MYB89	267	28815.03	9.85	59.91	-0.625
Os2R_MYB90	330	36061.25	6.26	48.71	-0.560
Os2R_MYB91	285	32783.44	5.60	54.26	-0.800
Os2R_MYB92	237	27226.09	8.82	71.15	-0.665
Os2R_MYB93	311	34063.18	5.59	57.25	-0.479
Os2R_MYB94	266	28647.88	8.48	65.22	-0.647
Os2R_MYB95	845	93326.61	5.17	66.41	-0.695
Os2R_MYB96	409	43722.43	6.33	59.81	-0.521
Os2R_MYB97	300	33864.46	5.37	50.19	-0.718
Os2R_MYB98	242	27843.81	8.39	67.38	-0.671
Os2R_MYB99	297	32057.24	8.86	35.28	-0.419
Average	324	35218.68	6.80	57.15	-0.536
