# Supplementary material for: Genome-Wide Identification of R2R3-MYB Transcription Factor and Expression Analysis under Abiotic Stress in Rice
Source: Plants (Basel). 2022 Jul 25;11(15):1928. doi: 10.3390/plants11151928 (PMC9330779; doi:10.3390/plants11151928)
Supplement: Supplementary file 1 [file plants-11-01928-s001.zip › Table S2 Statistical analysis of the intron numbers of 99 Os2R_MYB genes.pdf]

Table S2. Statistical Analysis of the Intron Numbers of 99 Os2R_MYB Genes

Name	Gene ID	Intron Numbers	Gene Numbers	Percentages
Os2R_MYB6	*LOC_Os01g19330.1*	0	10	10.10%
Os2R_MYB20	*LOC_Os01g74590.1*			
Os2R_MYB22	*LOC_Os02g09480.1*			
Os2R_MYB34	*LOC_Os03g13310.1*			
Os2R_MYB35	*LOC_Os03g19630.1*			
Os2R_MYB37	*LOC_Os03g26130.1*			
Os2R_MYB54	*LOC_Os05g28320.1*			
Os2R_MYB65	*LOC_Os06g14010.1*			
Os2R_MYB69	*LOC_Os07g25370.1*			
Os2R_MYB79	*LOC_Os08g34960.1*			
Os2R_MYB2	*LOC_Os01g04930.1*	1	24	24.24%
Os2R_MYB3	*LOC_Os01g07450.1*			
Os2R_MYB7	*LOC_Os01g19970.1*			
Os2R_MYB9	*LOC_Os01g45090.1*			
Os2R_MYB11	*LOC_Os01g50110.1*			
Os2R_MYB16	*LOC_Os01g63160.1*			
Os2R_MYB18	*LOC_Os01g65370.1*			
Os2R_MYB19	*LOC_Os01g74410.1*			
Os2R_MYB26	*LOC_Os02g41510.1*			
Os2R_MYB29	*LOC_Os02g46780.1*			
Os2R_MYB36	*LOC_Os03g20090.1*			
Os2R_MYB50	*LOC_Os04g50680.1*			
Os2R_MYB55	*LOC_Os05g35500.1*			
Os2R_MYB56	*LOC_Os05g37060.1*			
Os2R_MYB57	*LOC_Os05g37730.1*			
Os2R_MYB62	*LOC_Os06g02250.1*			
Os2R_MYB72	*LOC_Os07g43580.1*			
Os2R_MYB73	*LOC_Os07g48870.1*			
Os2R_MYB81	*LOC_Os08g43550.1*			
Os2R_MYB85	*LOC_Os09g36250.1*			
Os2R_MYB86	*LOC_Os09g36730.1*			
Os2R_MYB89	*LOC_Os11g10130.1*			
Os2R_MYB93	*LOC_Os12g03150.1*			
Os2R_MYB96	*LOC_Os12g33070.1*			
Os2R_MYB1	*LOC_Os01g03720.1*			
Os2R_MYB4	*LOC_Os01g09590.1*			
Os2R_MYB5	*LOC_Os01g18240.1*			
Os2R_MYB8	*LOC_Os01g36460.1*			
Os2R_MYB10	*LOC_Os01g49160.1*			
Os2R_MYB12	*LOC_Os01g50720.1*			
Os2R_MYB14	*LOC_Os01g52410.1*			
Os2R_MYB15	*LOC_Os01g59660.1*			

Os2R_MYB17	*LOC_Os01g63680.1*	2	59	59.60%
Os2R_MYB21	*LOC_Os02g02370.1*			
Os2R_MYB23	*LOC_Os02g17190.1*			
Os2R_MYB24	*LOC_Os02g36890.1*			
Os2R_MYB25	*LOC_Os02g40530.1*			
Os2R_MYB27	*LOC_Os02g42850.2*			
Os2R_MYB28	*LOC_Os02g42870.1*			
Os2R_MYB30	*LOC_Os02g49986.1*			
Os2R_MYB31	*LOC_Os02g51799.1*			
Os2R_MYB33	*LOC_Os03g04900.1*			
Os2R_MYB38	*LOC_Os03g27090.1*			
Os2R_MYB39	*LOC_Os03g29614.1*			
Os2R_MYB40	*LOC_Os03g38210.1*			
Os2R_MYB41	*LOC_Os03g51110.1*			
Os2R_MYB42	*LOC_Os03g56090.1*			
Os2R_MYB43	*LOC_Os04g38740.1*			
Os2R_MYB44	*LOC_Os04g39470.1*			
Os2R_MYB45	*LOC_Os04g42950.1*			
Os2R_MYB46	*LOC_Os04g43680.1*			
Os2R_MYB47	*LOC_Os04g45020.1*			
Os2R_MYB48	*LOC_Os04g45060.1*			
Os2R_MYB49	*LOC_Os04g46384.1*			
Os2R_MYB51	*LOC_Os04g50770.1*			
Os2R_MYB52	*LOC_Os05g04210.1*			
Os2R_MYB53	*LOC_Os05g04820.1*			
Os2R_MYB59	*LOC_Os05g46610.1*			
Os2R_MYB60	*LOC_Os05g48010.1*			
Os2R_MYB61	*LOC_Os05g49310.1*			
Os2R_MYB63	*LOC_Os06g10350.1*			
Os2R_MYB64	*LOC_Os06g11780.1*			
Os2R_MYB67	*LOC_Os07g12130.1*			
Os2R_MYB68	*LOC_Os07g14110.1*			
Os2R_MYB70	*LOC_Os07g31470.1*			
Os2R_MYB71	*LOC_Os07g37210.1*			
Os2R_MYB74	*LOC_Os08g05520.1*			
Os2R_MYB75	*LOC_Os08g15020.1*			
Os2R_MYB76	*LOC_Os08g33150.1*			
Os2R_MYB77	*LOC_Os08g33660.1*			
Os2R_MYB78	*LOC_Os08g33940.1*			
Os2R_MYB80	*LOC_Os08g37970.1*			
Os2R_MYB82	*LOC_Os09g23620.1*			
Os2R_MYB83	*LOC_Os09g24800.1*			
Os2R_MYB84	*LOC_Os09g26170.1*			

Os2R_MYB87	*LOC_Os10g33810.1*			
Os2R_MYB88	*LOC_Os10g35660.1*			
Os2R_MYB90	*LOC_Os11g35390.1*			
Os2R_MYB91	*LOC_Os11g45740.1*			
Os2R_MYB92	*LOC_Os11g47460.1*			
Os2R_MYB94	*LOC_Os12g07640.1*			
Os2R_MYB97	*LOC_Os12g37690.1*			
Os2R_MYB98	*LOC_Os12g37970.1*			
Os2R_MYB13	*LOC_Os01g51260.1*	3	2	2.02%
Os2R_MYB58	*LOC_Os05g41166.1*			
Os2R_MYB32	*LOC_Os02g54520.1*	5	2	2.02%
Os2R_MYB99	*LOC_Os12g41920.2*			
Os2R_MYB66	*LOC_Os06g40330.1*	6	1	1.01%
Os2R_MYB95	*LOC_Os12g13570.1*	7	1	1.01%
