# Supplementary material for: Genome-Wide Identification of R2R3-MYB Transcription Factor and Expression Analysis under Abiotic Stress in Rice
Source: Plants (Basel). 2022 Jul 25;11(15):1928. doi: 10.3390/plants11151928 (PMC9330779; doi:10.3390/plants11151928)
Supplement: Supplementary file 1 [file plants-11-01928-s001.zip › Table S6 Statistical analysis of Os2R_MYB genes expression level under the PEG and CdCl2 stress.pdf]

Table S6. Statistical Analysis of Os2R_MYB Genes Expression Level under the PEG and $CdCl_2$ Stresses

Stresses	Gene Name	Gene ID	Expression patterns
PEG	*Os2R_MYB36*	*LOC_Os03g20090.1*	up-regulated
	Os2R_MYB1	*LOC_Os01g03720.1*	
	Os2R_MYB18	*LOC_Os01g65370.1*	
	Os2R_MYB81	*LOC_Os08g43550.1*	
	Os2R_MYB98	*LOC_Os12g37970.1*	
	Os2R_MYB3	*LOC_Os01g07450.1*	down-regulated
	Os2R_MYB7	*LOC_Os01g19970.1*	
	Os2R_MYB15	*LOC_Os01g59660.1*	
	Os2R_MYB16	*LOC_Os01g63160.1*	
	Os2R_MYB21	*LOC_Os02g02370.1*	
	Os2R_MYB23	*LOC_Os02g17190.1*	
	Os2R_MYB47	*LOC_Os04g45020.1*	
	Os2R_MYB51	*LOC_Os04g50770.1*	
	Os2R_MYB56	*LOC_Os05g37060.1*	
	Os2R_MYB76	*LOC_Os08g33150.1*	
	Os2R_MYB89	*LOC_Os11g10130.1*	
	Os2R_MYB93	*LOC_Os12g03150.1*	
	Os2R_MYB2	*LOC_Os01g04930.1*	
	Os2R_MYB90	*LOC_Os11g35390.1*	
	Os2R_MYB99	*LOC_Os12g41920.2*	
$CdCl_2$	*Os2R_MYB36*	*LOC_Os03g20090.1*	up-regulated
	Os2R_MYB1	*LOC_Os01g03720.1*	down-regulated
	Os2R_MYB18	*LOC_Os01g65370.1*	
	Os2R_MYB81	*LOC_Os08g43550.1*	
	Os2R_MYB98	*LOC_Os12g37970.1*	
	Os2R_MYB3	*LOC_Os01g07450.1*	
	Os2R_MYB7	*LOC_Os01g19970.1*	
	Os2R_MYB15	*LOC_Os01g59660.1*	
	Os2R_MYB16	*LOC_Os01g63160.1*	
	Os2R_MYB21	*LOC_Os02g02370.1*	
	Os2R_MYB23	*LOC_Os02g17190.1*	
	Os2R_MYB47	*LOC_Os04g45020.1*	
	Os2R_MYB51	*LOC_Os04g50770.1*	
	Os2R_MYB56	*LOC_Os05g37060.1*	
	Os2R_MYB76	*LOC_Os08g33150.1*	
	Os2R_MYB89	*LOC_Os11g10130.1*	
	Os2R_MYB93	*LOC_Os12g03150.1*	
	Os2R_MYB2	*LOC_Os01g04930.1*	had no obvious changes
	Os2R_MYB90	*LOC_Os11g35390.1*	
	Os2R_MYB99	*LOC_Os12g41920.2*	
PEG and $CdCl_2$	*Os2R_MYB36*	*LOC_Os03g20090.1*	up-regulated
	Os2R_MYB1	*LOC_Os01g03720.1*	expression pattern opposite
	Os2R_MYB18	*LOC_Os01g65370.1*	
	Os2R_MYB81	*LOC_Os08g43550.1*	
	Os2R_MYB98	*LOC_Os12g37970.1*	
