# Supplementary material for: Genome-Wide Identification of R2R3-MYB Transcription Factor and Expression Analysis under Abiotic Stress in Rice
Source: Plants (Basel). 2022 Jul 25;11(15):1928. doi: 10.3390/plants11151928 (PMC9330779; doi:10.3390/plants11151928)
Supplement: Supplementary file 1 [file plants-11-01928-s001.zip › Table S7 Quantitative RT-PCR Primers.pdf]

Table S7. Quantitative RT-PCR Primers

Quantitative RT-PCR Primers	
Name	Sequence (5’-3’)
qRT-Os2R_MYB1-F	GCTAAGATGGCTCAACTA
qRT-Os2R_MYB1-R	CCTCCAGTAGTTCTTGAT
qRT-Os2R_MYB2-F	GGACTGAAGAAATATGGG
qRT-Os2R_MYB2-R	GATGTGTTGGACTGATTG
qRT-Os2R_MYB3-F	GACAACGAGATCAAGAAC
qRT-Os2R_MYB3-R	GTGAGAGAAGGTGTTGAA
qRT-Os2R_MYB7-F	GACATCATCCTCAACCTC
qRT-Os2R_MYB7-R	CGAGTTCCAGTAGTTCTT
qRT-Os2R_MYB15-F	TACCCATCTATCCTACCA
qRT-Os2R_MYB15-R	CCAAGGAGATTGCTTATAG
qRT-Os2R_MYB16-F	GTGGTGTTATGAAGAGAG
qRT-Os2R_MYB16-R	CAGGTCCTACATTGTATG
qRT-Os2R_MYB18-F	CTCATCATCAAGCTCCAC
qRT-Os2R_MYB18-R	CGTGTTCCAGTAGTTCTT
qRT-Os2R_MYB21-F	GGTGAAGAACTACTGGAA
qRT-Os2R_MYB21-R	GTGATGATGATGGTGATG
qRT-Os2R_MYB23-F	CATAACAACTGGGAAACTG
qRT-Os2R_MYB23-R	GCCATTCTACACCACTTA
qRT-Os2R_MYB36-F	CTGCTCGTCAACTACATC
qRT-Os2R_MYB36-R	GTTCTTGATCTCGTTGTC
qRT-Os2R_MYB47-F	GAGATGGATCAATTACCTG
qRT-Os2R_MYB47-R	GTTCCAGTAGTTCTTCAC
qRT-Os2R_MYB51-F	GTCGCCTACATTCAGAAG
qRT-Os2R_MYB51-R	CTTGATGATGGTCTCCTC
qRT-Os2R_MYB56-F	AGGGCTAGAGAAATATGG
qRT-Os2R_MYB56-R	CTGTCGGATGAAGAACTT
qRT-Os2R_MYB76-F	GGCAATAGGTGGTCTAAG
qRT-Os2R_MYB76-R	CCGTGTTCTTTCTCTCTG
qRT-Os2R_MYB81-F	GACGACCTCATCATCAAG
qRT-Os2R_MYB81-R	CGTGTTCCAGTAGTTCTT
qRT-Os2R_MYB89-F	AATAGCTCGCTATGCTTC
qRT-Os2R_MYB89-R	GTACCGTTAAACGACTTG
qRT-Os2R_MYB90-F	CAAGAAGAAGGTGAAGAG
qRT-Os2R_MYB90-R	GTTGTTAGGGTTGTAGTAG
qRT-Os2R_MYB93-F	CAACTCCACTTCATCCTC
qRT-Os2R_MYB93-R	GAGATGTTGTCCATGCTG
qRT-Os2R_MYB98-F	AGTGAGAGAAGAGATACG
qRT-Os2R_MYB98-R	CCTGATACTTTGGCAATG
qRT-Os2R_MYB99-F	CAGATGGTGATGATGATG
qRT-Os2R_MYB99-R	CTATGGCTGTCTTGTATG
OsActin1-F	CCCTCCTGAAAGGAAGTACAGTGT
OsActin1-R	ACTCGTCGTACTCAGCCTTGG
OsGAPDH-F	GGGCTGCTAGCTTCAACATC
OsGAPDH-R	TTGATTGCAGCCTTGATCTG
